# α-Al_2_O_3_ Functionalized with Lithium Ions Especially Useful as Inert Catalyst Bed Supports

**DOI:** 10.3390/molecules30030577

**Published:** 2025-01-27

**Authors:** Mirjana Stamenić, Timotei Bogdan Bacoș, Aleksandar Milivojević, Vuk Adžić, Mihaela Ciopec, Nicoleta Sorina Nemeş, Adina Negrea, Adrian Eugen Cioablă

**Affiliations:** 1 Faculty of Mechanical Engineering, University of Belgrade, Kraljice Marije 16, 11120 Belgrade, Serbia; mstamenic@mas.bg.ac.rs (M.S.); amilivojevic@mas.bg.ac.rs (A.M.); vadzic@mas.bg.ac.rs (V.A.); 2 Mechanical Engineering Faculty, Politehnica University Timisoara, Mihai Viteazu Blv., 1, 300222 Timisoara, Romania; adrian.cioabla@upt.ro; 3 Research Institute for Renewable Energies—ICER, Politehnica University Timisoara, 138 Gavril Musicescu Street, 300501 Timisoara, Romania; mihaela.ciopec@upt.ro (M.C.); adina.negrea@upt.ro (A.N.); 4 Faculty of Chemical Engineering, Biotechnologies and Environmental Protection, Politehnica University Timisoara, Victoriei Square, No. 2, 300006 Timisoara, Romania; nicoleta.nemes@upt.ro

**Keywords:** tabular alumina balls, lithium, catalyst, absorbent, PIM burner, combustion stability

## Abstract

The alumina, in the form of α-Al_2_O_3_ tabular balls, considered in this study is a high-purity form of aluminum oxide that has been fired at high temperatures (well above 1900 °C), virtually removing porosity. However, the purity and inertness of the surface of the Al_2_O_3_ tabular balls minimize the catalytic activity, which is why lithium doping was tried. Thus, the target of this study was the effect of doping with lithium ions in some tabular balls of Al_2_O_3_ (the crystalline structure is corundum) on the improvement of the catalytic properties of alumina. This study examined the impact of a lithium catalyst on the combustion of various fuels within a porous inert medium (PIM) burner. This study specifically compared low calorific gaseous fuel (e.g., biogas) combustion in a PIM burner with and without the lithium catalyst. The experimental setup comprised a gas preparation unit for mixing CNG and CO_2_ to simulate biogas and a PIM burner. The PIM burner comprised Al_2_O_3_ spheres (13 mm diameter, 45% porosity) in a random packing configuration. Three fuels, varying in composition and lower heating value (LHV ranging from 20.771 to 27.695 MJ/m^3^), were combusted at air ratios ranging from 1.67 to 1.79. The results indicated that the catalyst increased peak combustion temperatures by 23.2 °C to 51.4 °C, depending on the fuel type and air ratio. Significantly higher carbon monoxide (CO) concentrations were observed without the catalyst, particularly with fuel type F1, while nitrous oxide (NOx) levels remained consistently low. Upstream flame propagation was observed in the presence of the catalyst. These findings demonstrate the potential of lithium catalysts to enhance combustion stability and reduce emissions in porous media combustion burners. Following these studies, it can be stated that Li(I) has the role of promoter of the catalytic process.

## 1. Introduction

Porous media combustion represents a significant advancement in combustion technology. PIM burners offer advantages over conventional open-flame burners. These burners have higher flame stability, enhanced combustion efficiency, better control over the combustion process, and lower temperatures in the combustion zone [1,2]. Spherical particle packed beds are frequently used in experimental porous burner designs [3,4,5]. Methane served as the primary fuel in studies [1,5,6], while a study [7] utilized liquefied petroleum gas (LPG). The combustion technology in a porous structure enables the formation of a combustion chamber, which extends the range of applications. The porous medium effectively increases the stability of the flame and thus enables a reduction in the dimensions of the appliance itself. Hauwel et al. [8] demonstrated that catalytic-free combustion of hydrocarbon fuels in inert porous media can achieve stable combustion at significantly higher flow rates and beyond typical free-space flammability limits. This is attributed to efficient heat exchange within the porous structure between the combustion products and the incoming fuel–air mixture. A dual-layer ceramic foam porous burner’s stable operating limits were determined in [9] by varying the air/fuel ratio (1.3 < λ < 1.55) and power density (up to 4000 kW/m^2^) during methane combustion. Stable flames were observed at velocities exceeding laminar burning velocities, with CO and NOx emissions below 15 ppm and 10 ppm, respectively (NOx across all measurements, CO below 2000 kW/m^2^ power density).

The most important performance parameters of burners in general and of burners with combustion in a porous structure, on the basis of which the calculation, i.e., the selection, is made, are the following:

- Heat output;
- Type of fuel;
- Stability of operation;
- Emission of components harmful to the environment;
- Material of the burner;
- Heat transfer mechanism.

It is a well-known fact that the activity and selectivity of a catalytic material can be modified using different methods like doping on a fine divided support [3,4,5] of certain metallic oxides [6,7,8,9,10,11,12].

The catalytic activity of lithium could be modified by loading on solid supports, such as alumina or silica [3,13,14,15,16,17,18].

Alumina is an important industrial chemical substance that has wide applications [19,20,21,22]. In particular, the class of aluminum oxides, known as “transitional aluminas”, plays an important commercial role in many chemical processes [19,20,21,22]. The large applications of alumina in catalytic processes and adsorption processes can be attributed to a combination of favorable textural properties, such as the appropriate size of the pore distribution and large area, and chemical properties that can be either acidic or basic depending on the transition structure of the alumina [19,20,21,22].

Those elements can contribute to an effect that changes the degree of dispersion of the metal ion with which the surface of the support is doped. However, the role of the support in the catalytic reaction is not always inert. In some cases, the interaction of the support with the metal ion is so important that it has been called strong metal support interaction (SMSI) [3,23,24].

The doping process produces significant changes in the surface of the support as a result of the interaction with the metal ion, activating the catalytic activity of the support depending on the amount of deposited metal ion [3,25,26]. The present investigation was dedicated to the study of the effect of lithium ion doping of tabular α-Al_2_O_3_ balls (the crystalline structure is corundum). Alumina is a high-purity form of aluminum oxide that has been fired at high temperatures (well above 1900 °C), but the purity and inertness of the surface of the α-Al_2_O_3_ tabular balls minimize the catalytic activity, which is why lithium doping was tested. In regards to the existing literature, the present study describes the development of a small-scale pilot test rig used for testing the influence of a Li-based catalyst for different testing of flammable mixture gases (similar to biogas-based recipes), excluding the hydrogen sulphide component for determining the impact on CO and Nox emissions.

## 2. Results and Discussion

### 2.1. Li/Al_2_O_3_ Material Characterization

**Figure 1:**
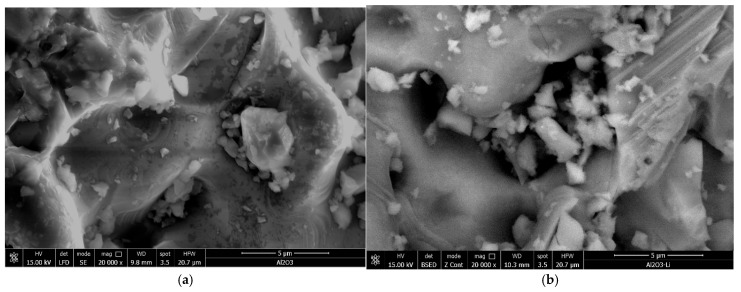
Scanning electron microscopy, SEM; magnification 20,000×. (**a**) Al_2_O_3_, (**b**) Li/Al_2_O_3_.

Scanning electron microscopy (SEM) was used to analyze the surface morphology of the Li/Al_2_O_3_ material before (Figure 1a) and after doping with Li(I) ions (Figure 1b).

**Figure 2:**
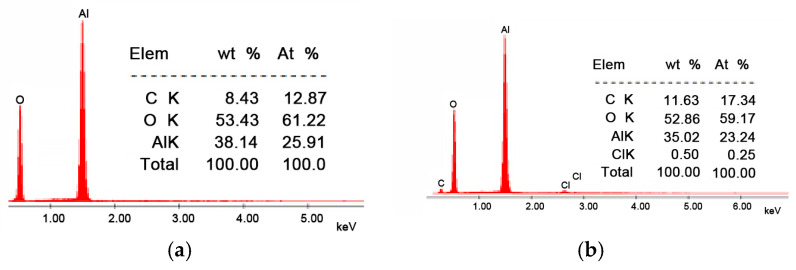
Energy-dispersive X-ray spectroscopy, EDX. (**a**) Al_2_O_3_, (**b**) Li/Al_2_O_3_.

From the SEM images presented in Figure 1, it can be observed that the morphology of the Li/Al_2_O_3_ material after functionalization changes but insignificantly. This statement is supported by the EDX spectra (Figure 2).

In the EDX spectrum of the Li/Al_2_O_3_ material, the presence of the specific chlorine peak is observed, which confirms the doping of the Al_2_O_3_ tabular balls surface with Li(I) ions (from the LiCl solution). This fact is also observed from the quantitative data presented (0.50%, mass percentage).

### 2.2. Al_2_O_3_ Doping Studies

In order to improve the catalytic properties of Al_2_O_3_ tabular balls by doping its surface with lithium ions, the maximum amount of Li(I) ions with which the Al_2_O_3_ tabular balls’ surface can be loaded was determined. For this, it was necessary to determine the size of the tabular alumina balls, the optimal pH of Li(I) ions, and the optimal contact time and temperature for which the maximum adsorption capacity of Al_2_O_3_ tabular balls is obtained.

**Figure 3:**
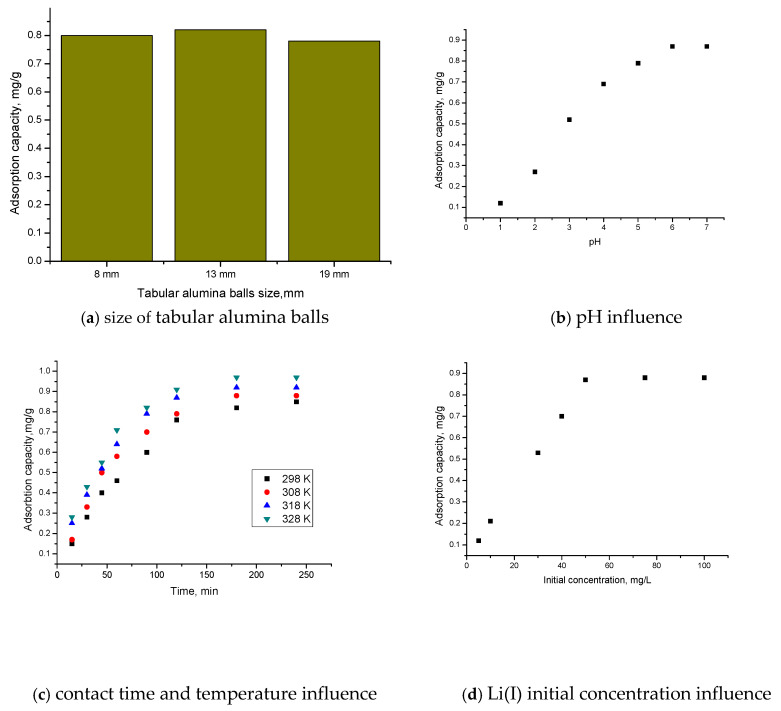
The dependences of the adsorption capacity on size of tabular alumina balls (**a**); pH (**b**); contact time and temperature (**c**); and initial Li(I) concentration (**d**).

Figure 3 shows the dependences of the adsorption capacity of tabular alumina balls on size (a), pH (b), contact time and temperature (c), and initial Li(I) concentration (d).

From Figure 3a, it is observed that the size of the tabular alumina balls does not greatly influence the adsorption capacity. For a tabular alumina balls’ size of 13 mm, the adsorption capacity (0.83 mg Li(I)/g Al_2_O_3_ tabular balls) is slightly higher than for a tabular alumina balls’ size of 8 mm or 19 mm. The dependence of pH, contact time, and initial concentration of Li(I) on adsorption capacity was determined for a tabular alumina balls’ size of 13 mm.

It was found that, with the increase in pH (Figure 3b), the pH also increases until pH~6, after which the adsorption capacity remains constant (~0.87 mg Li(I)/g Al_2_O_3_ tabular balls). As the contact time increases (Figure 3c), the adsorption capacity of the material increases up to a contact time of 120 min, after which it remains constant (~0.82 mg Li(I)/g Al_2_O_3_ tabular balls).

Also, with the increase in temperature, the adsorption capacity increases but insignificantly, for which the subsequent studies are carried out at 298 K.

**Figure 4:**
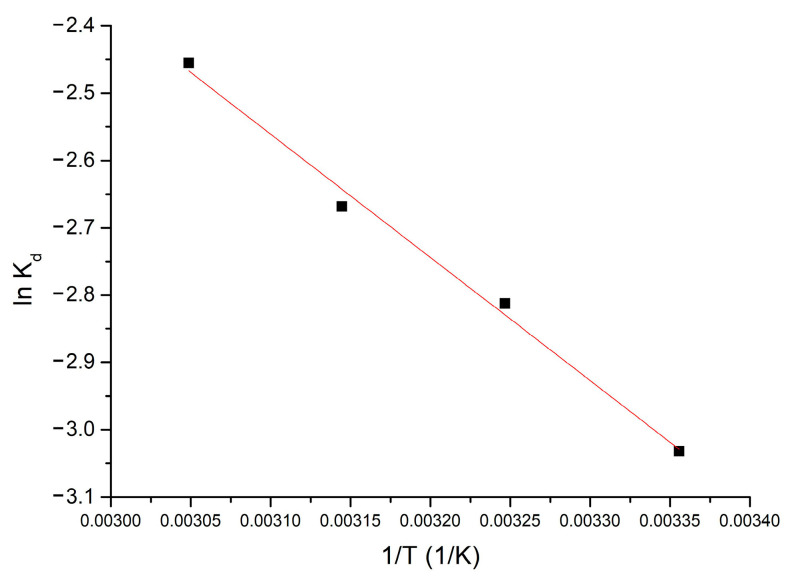
Thermodynamic studies for 298–328 K interval (influence of the equilibrium constant, *K*_d_, versus 1/T).

Thermodynamic studies were performed in the temperature range 298–328 K. The Gibbs free energy value was calculated using the Gibbs–Helmholtz equation. Using the van’t Hoff equation and from the equation of the line obtained from the graphical representation of ln *K_d_* = f(1/*T*), according to Figure 4, the standard variation of entropy ΔS° and the standard variation of enthalpy ΔH° can be calculated.

Table 1 shows the thermodynamic parameters obtained at the four temperatures.

From the resulting data, it is observed that ΔH° has a positive value (15.22 kJ mol^−1^), which means that the adsorption process is endotherm. The affinity shown by the Al_2_O_3_ tabular balls towards the Li(I) is highlighted by the appearance of electrostatic interactions, being an endothermic process. Because ΔH° < 50 kJ mol^−1^, the process is considered to be physical adsorption [27].

Due to the fact ΔG° has negative values the process is spontaneous. Because ΔG° various with temperature (increases, absolute value, between 298–328 K), indicates that the adsorption process is influenced by temperature. The fact that the ΔS° value is positive (174.3 J mol^−1^ k^−1^) indicates that the adsorption process is favored, occurring at the interface of the Al_2_O_3_ material/solution with Li(I).

Thus, the optimal conditions for doping Li(I) are pH~6, contact time 120 min, and temperature 298 K for a tabular alumina balls’ size of 13 mm. Under these conditions, the maximum adsorption/doping capacity of the material with Li(I) ions is 0.88 mg Li(I)/g Al_2_O_3_ tabular balls. To obtain this maximum adsorption capacity, an initial concentration of 75 mg Li(I)/L is required. For functionalization, Al_2_O_3_ tabular balls and Li(I) ions were brought into contact. The obtained material was then dried for 24 h at a temperature of 323 K.

### 2.3. Experimental Firing Tests

The burner’s thermal power was set to 1.5 kW for all experimental tests. Table 2 details the composition of the compressed natural gas (CNG) used in these experiments.

Different ratios of CNG and CO_2_ were mixed to simulate varying qualities of gaseous fuels. These mixtures were tested both with and without a lithium catalyst. The catalyst was applied as a coating on the alumina balls, serving as a catalyst carrier. Table 3 presents the fuel compositions for each CNG/CO_2_ ratio.

Combustion conditions, including air ratio and burner power output, were consistent for each fuel type (Table 4).

**Figure 5:**
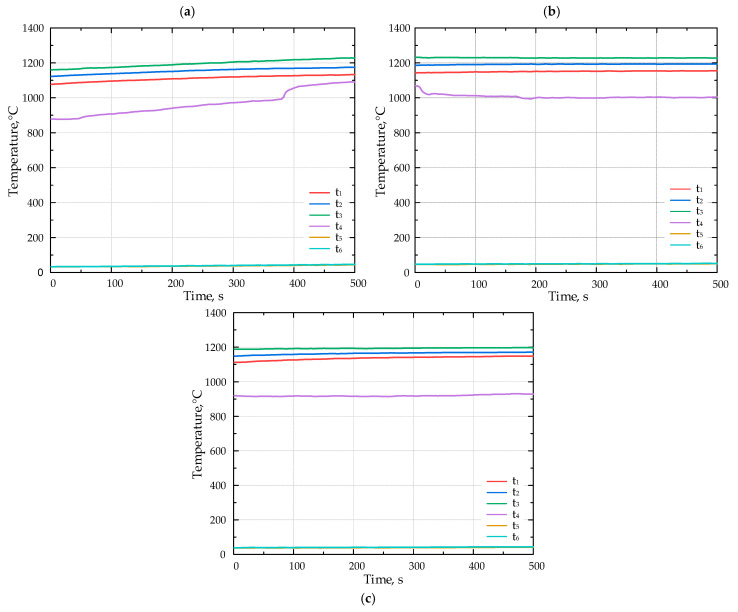
The temperature inside the porous medium for different fuels (**a**) F1, (**b**) F2, and (**c**) F3 without catalyst.

**Figure 6:**
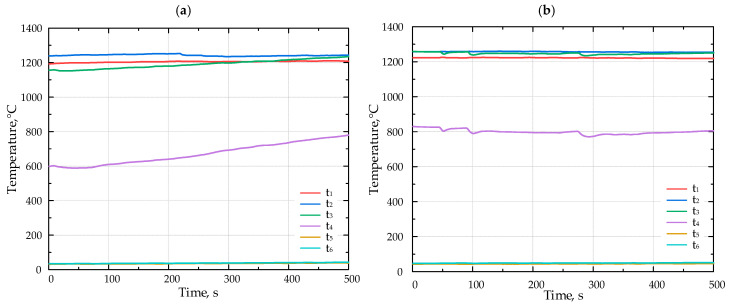

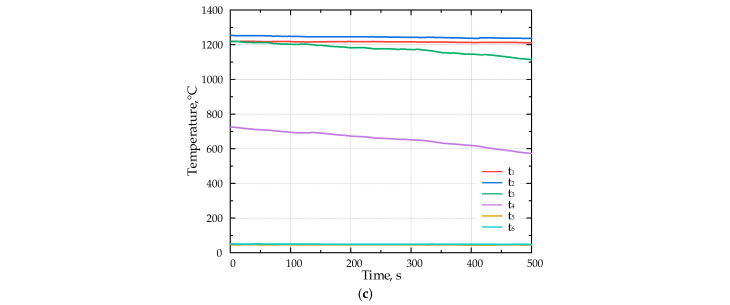
The temperature inside the porous medium for different fuels (**a**) F1, (**b**) F2, and (**c**) F3 with Li-catalyst.

The lithium catalyst was coated onto tabular alumina balls (Al_2_O_3_), matching the size of the balls (*d* = 13 mm) composing the porous inert media (PIM). This ensured that the PIM’s porosity remained unchanged (*ε* = 0.45). Figure 5 presents the results of the combustion experiments for various fuels without the catalyst. The duration of temperature recordings ranged from 550 to 900 s; however, the x-axis on the diagrams presented in Figure 5 and Figure 6 displays a time span between 0 and 500 s.

Figure 6 presents the results of the combustion experiments for various fuels with the lithium catalyst.

**Figure 7:**
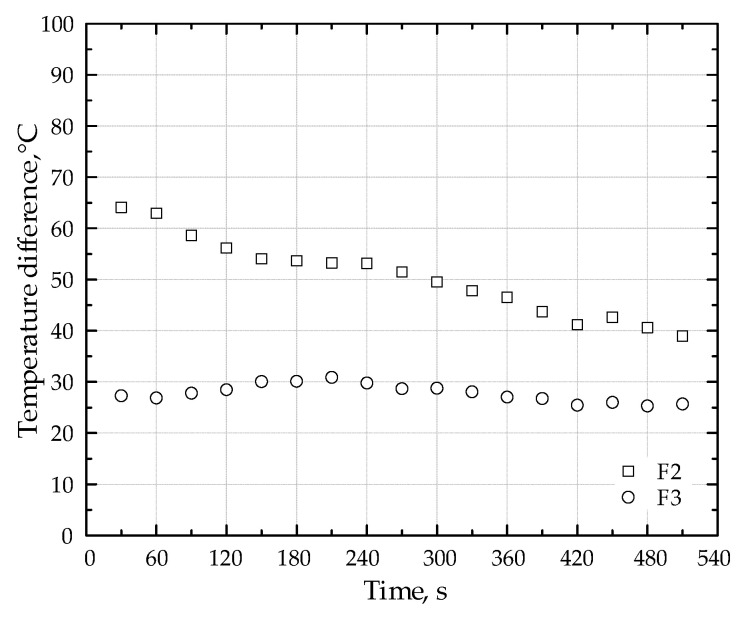
Effect of Li-Catalyst on peak temperature differences in porous media: fuels F2 and F3.

Table 5 presents a comparison of maximum temperatures within the porous medium as well as average CO and NOx concentrations for burners both with and without the lithium catalyst. These data are shown for three different fuel types. Figure 7 presents a comparative analysis of the temperature difference at the point of peak temperature within the porous medium. This analysis contrasts the temperature profiles obtained with and without the addition of the Li-catalyst, specifically for fuel types F2 and F3. The figure facilitates a direct visualization of the catalytic impact on the maximum temperature attained within the porous media while combusting different fuels (F2 and F3).

Introducing the Li-catalyst shifted the flame position upstream. During the experiments with the catalyst, the flame moved from the position of the thermocouple t_3_ to t_2_.

Following the above presented material, we can state that Li(I) has the role of promoter of the catalytic process.

## 3. Materials and Methods

In this work, it was aimed to improve the catalytic properties of Al_2_O_3_ by doping its surface with lithium ions. The doping was carried out by functionalization by impregnation, using the SIR (solvent impregnated resin) method [28].

According to the technical chart [29], the tabular alumina balls (Almatis Ltd., Iwakuni, Japan) contained 99.7% Al_2_O_3_, and the dimensions taken in the study were 8 mm, 13 mm, and 19 mm.

To obtain the Li/Al_2_O_3_ material, a LiCl solution (Sigma–Aldrich, Merck, St. Louis, MI, USA) was contacted with Al_2_O_3_ (alumina tabular balls contain 99.7% Al_2_O_3_) under the optimal conditions that will be established later. For functionalization, Al_2_O_3_ and Li(I) ions were brought into contact. The material obtained was then dried for 24 h at a temperature of 323 K.

The obtained material was characterized by scanning electron microscopy (SEM) and X-ray energy dispersive (EDX) using the X-ray energy dispersive spectrometer, FEI Quanta FEG 250 instrument (FEI, Hillsbro, OR, USA). The images were processed in the low-vacuum system using an LFD detector (FEI Company, Hillsboro, OR, USA) to prevent the shadowing effect of the particles. The accelerating voltage used for the sample’s irradiation was between 15 kV, with a spot size of 3.5 and a pressure of 90 Pa. The free working distance (FWD) was around 10 mm.

In order to establish the maximum amount of Li(I) with which Al_2_O_3_ can be loaded, a series of studies were carried out. Thus, the influence of some parameters (size of tabular alumina balls, pH, contact time, temperature, and the initial concentration of Li(I) ions) on the adsorption capacity of the Al_2_O_3_ tabular balls was studied.

In order to determine the influence of the size of the tabular alumina balls on the adsorption capacity of the material, ~1 g of tabular alumina balls of different sizes (8, 13, and 19 mm) was weighed, over which 25 mL of a solution with a concentration of *C*_0_ = 50 mg Li(I)/L was added at a contact time of 60 min, pH = 6, and temperature of 298 K.

The influence of the pH of the solutions is related to the species of Li(I) ion present in the solution. Thus, the pH varied in the range 1–7 at an initial concentration of *C*_0_ = 50 mg Li(I)/L, 1 g material, 25 mL solution, contact time 1 h, temperature 298 K, and alumina balls’ size of 13 mm. The pH of the solution was measured using the METTLER TOLEDO SevenCompact pH meter (METTLER TOLEDO, Greifensee, Switzerland). The pH was not varied above pH = 7 because lithium precipitates around pH~8.

In order to determine the influence of the contact time and temperature on the adsorption capacity of the Al_2_O_3_ tabular balls, 1 g of tabular alumina balls was accurately weighed, over which 25 mL of a solution with a concentration of *C*_0_ = 50 mg Li(I)/L was added. The samples were stirred for different times (15, 30, 45, 60, 120, 180, and 240 min) in a Julabo (Seelbach, Germany) SW23 water bath at different temperature (298, 308, 318, and 328 K), pH~6, and alumina balls’ size of 13 mm. The samples were stirred at 200 rpm.

To establish the effect of the initial concentration of lithium ions on the adsorption capacity of the Al_2_O_3_ tabular balls but also to establish the maximum amount of lithium that the material can adsorb, Li(I) solutions of different initial concentrations (5, 10, 30, 50, 75, and 100 mg/L) were prepared. These were obtained by appropriate dilution from a 1000 mg/L LiCl stock solution. Adsorption was carried out at pH = 6 for 120 min at a temperature of 298 K and an alumina balls’ size of 13 mm. The residual concentration of Li(I) ions was measured by atomic absorption spectroscopy using a Varian AAS FS280 atomic absorption spectrometer (Agilent Technologies, Palo Alto, CA, USA).

The adsorption capacity of the used material was calculated using the following equation:

$$q=\frac{\left(C_{0}-C_{f}\right)V}{m}$$

where *C*_0_—initial Li(I) concentration from solution, (mg/L);

*C_f_*—residual Li(I) concentration from solution, (mg/L);

*V*—volume of solution, (L);

*m*—mass of adsorbant material, (g).

To elucidate the adsorption mechanism, using the Gibbs–Helmholtz equation, the value of the Gibbs free energy is calculated [30]:

∆G°=∆H°−T∆S°

where Δ*G*°—free Gibbs energy standard variation (kJ mol^−1^);

Δ*H*°—enthalpy standard variation (kJ mol^−1^);

Δ*S*°—entropy standard variation (J mol^−1^ k^−1^);

*T*—absolute temperature (K).

Using the van’t Hoff equation, the standard enthalpy and entropy values associated with the adsorption process are determined [31,32]. The two parameters are obtained from the slope of the line, respectively, from the ordinate at the origin of the linear dependence between ln *K_d_* and 1/*T*:

$$\ln K_{d}=\frac{∆S{}^{\circ}}{R}-\frac{∆H{}^{\circ}}{RT}$$

where *K_d_*—equilibrium constant;

Δ*S*°—entropy standard variation (J mol^−1^ k^−1^);

Δ*H*°—enthalpy standard variation (kJ mol^−1^);

*T*—absolute temperature (K);

*R*—ideal gas constant (8314 J mol^−1^ K^−1^).

The equilibrium constant of the adsorption process is the ratio of the adsorption capacity at equilibrium, *q_e_*, to the equilibrium concentration, *C_e_*:

$$K_{d}=\frac{q_{e}}{C_{e}}$$

The energy required to bring the adsorbate into contact with the adsorbate surface is represented by the positive value of the standard enthalpy (Δ*H*°).

The negative value of the Gibbs free energy variation, Δ*G*°, obtained from the experimental data, indicates that the adsorption process is a spontaneous and natural process.

The speed of the adsorption process at the adsorbent/solution interface is given by the positive value of the entropy change in the adsorption process, Δ*S*°.

Lithium plays a significant role as a catalyst in combustion processes, especially in chemical reactions involving organic fuels. Here are some important aspects related to the use of lithium: (i) improvement of combustion efficiency (lithium can contribute to increasing the efficiency of combustion processes by reducing the temperatures required to initiate combustion reactions); (ii) emission control (the use of lithium in catalysts can help reduce emissions of toxic gases and pollutants such as NOx); (iii) stabilization of free radicals (lithium can interact with free radicals produced during combustion, stabilizing them and contributing to more complete combustion); (iv) application in hydrocarbon-based fuels (in particular, lithium is used in catalysts for oil and natural gas, where it can help decompose complex organic compounds); (v) effects on combustion particles (lithium can influence the formation of particles in combustion products, contributing to the formation of smaller particles, which are easier to control and less harmful) [33,34,35].

The role of lithium ions as a promoter of aluminum oxide is described by the following:

1. Catalytic Activity Enhancement: Lithium ions can be introduced into aluminum oxide to modify its surface properties, which improves its affinity for reactants and enhances catalytic activity. These modifications can lead to more effective catalytic cycles in combustion reactions.
2. Stabilization of Active Sites: Lithium ions can stabilize active sites on aluminum oxide, facilitating better adsorption of reaction intermediates. This stabilization can optimize the overall catalytic performance during combustion processes.
3. Influence on Reaction Kinetics: Lithium ions may alter the kinetics of the reactions taking place over aluminum oxide, resulting in improved efficiency of combustion reactions. The presence of lithium can lead to enhanced transformation pathways that are beneficial for catalytic processes.

Determining Optimal Lithium Content:

Determining the optimal lithium content as a promoter of combustion catalysts typically involves systematic studies where various lithium concentrations are tested to evaluate their impact on catalytic performance. This includes assessing temperature profiles, reaction rates, and product distributions under defined conditions. The optimal lithium content would be the concentration at which catalytic performance is maximized without negative side effects, such as deactivation or reduced selectivity [36,37,38,39,40].

### 3.1. Experimental Installation

**Figure 8:**
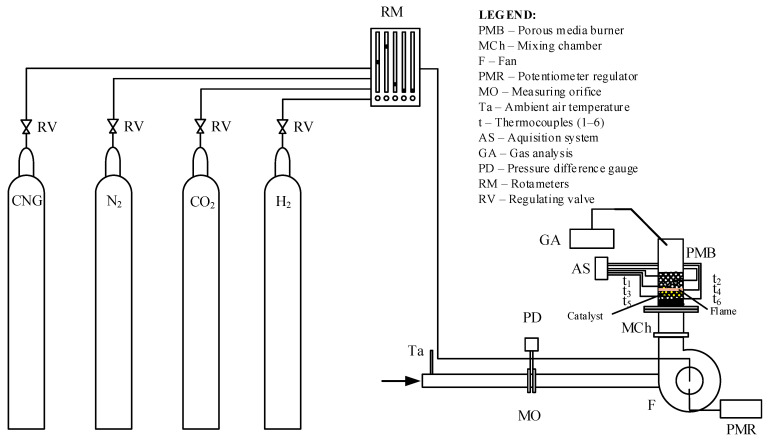
Experimental installation (the flame is marked with orange color and the Alumina balls with catalyst are marked with yellow color).

In the specific case of this test, the basic elements of the experimental installation used in this test are shown in Figure 8.

The porous inert media burner had a 65 mm inlet diameter and a 96 mm bulk material height.

### 3.2. The Procedure for the Experimental Tests

As shown in Figure 8, combustible gases are fed from the gas tanks with CNG, N_2_, H_2_, and CO_2_ into the mixing device with flowmeters integrated with control valves (RM). The gas mixture produced in this way simulates the composition of a low-calorie gaseous fuel. Only a mixture of CNG and CO_2_ was introduced during the first tests. Later, hydrogen was introduced as an enrichment and stabilizer component of the burning mixture, but this paper will not address the enrichment of low-calorific gaseous fuel with hydrogen. Further, the gaseous fuel produced in this way is mixed with the air fed from the immediate surroundings through the intake nozzle into the fan’s impeller, where the newly formed gas mixture is additionally homogenized. The airflow is controlled and varied by changing the number of revolutions of the fan impeller with a frequency controller (PMR). The airflow is measured using a measuring orifice (MO). The fan is located before the mixing chamber of the porous ceramic burner (MCh). Thermocouples (t_1_–t_6_) placed along the axis inside the burner’s porous structure are used to determine the temperature field. The composition of the combustion products was measured at the outlet of the burner by using the gas analyzer (GA).

The measurements were carried out with a mixture of gaseous fuel without hydrogen; then, a suitable catalyst was added to the burner at the position shown in Figure 1. The position of the catalyst corresponds to the immediate proximity of the flame zone.

## 4. Conclusions

This study investigates the effect of lithium ion doping on the catalytic properties of tabular α-Al_2_O_3_ (corundum crystalline structure).

To obtain the Li/Al_2_O_3_ material, a LiCl solution was contacted with the alumina tabular balls for functionalization in the optimal conditions established (pH~6, contact time 120 min, temperature 298 K, tabular alumina balls’ size 13 mm, 75 mg Li(I)/L). The obtained material was then dried for 24 h at a temperature of 323 K. The obtained material was characterized by scanning electron microscopy (SEM). The micrograph does not differ substantially, but the chlorine peak from the EDX spectra reveals functionalization.

The laboratory experiments of combustion of the biogas-like gaseous fuels in the ceramic porous burner with and without catalysts showed the following:

- Using fuel type F1 with a burner output of 1.5 kW and an air ratio of 1.67, the porous inert media (PIM) with the lithium catalyst reached a maximum temperature 23.2 °C higher than without the catalyst. For fuel types F2 and F3 with air ratios of 1.77 and 1.79, respectively, the PIM with the catalyst achieved temperature increases of 28.2 °C and 51.4 °C, respectively.
- Carbon monoxide (CO) concentrations were consistently higher in the PIM without the lithium catalyst, with the largest difference observed during the combustion of fuel type F1. Nitrogen oxide (NOx) concentrations remained stable and low throughout all experiments.
- The presence of the lithium catalyst shifted the flame position upstream.
- There is a substantial potential of lithium catalysts to improve combustion efficiency and reduce emissions in porous media applications.
- Further investigations are to be made for different positions of the catalyst in regard to the flue gas in order to improve the residence time between the flue gas and the catalytic material for better results in terms of further reducing the content of NOx and CO in the resulting flue gas from the combustion process.

## Figures and Tables

**Table 1:** Thermodynamic parameters for adsorption of Li (I) onto Al_2_O_3_.

Δ*H*° (kJ mol^−1^)	Δ*S*° (J mol^−1^ K^−1^)	Δ*G*° (kJ mol^−1^)				R^2^
15.22	174.3	298 K	308 K	318 K	328K	0.9907
		−51.9	−53.6	−55.4	−57.1	

**Table 2:** Composition of the compressed natural gas (CNG).

Component	CH_4_	C_2_H_6_	C_3_H_8_	CO_2_	N_2_	Hd, MJ/m^3^
(% mol)	93.69	1.11	0.31	0.35	4.54	34.6

**Table 3:** Fuel compositions for each CNG/CO_2_ ratio.

P, kW	Fuel Label	CNG:CO_2_	Hd, kJ/m^3^	Fuel Composition (% mol)
1.5	F1	80:20	27.695	CH_4_—74.95 C_2_H_6_—0.89 C_3_H_8_—0.25 CO_2_—20.28 N_2_—3.63
	F2	70:30	24.233	CH_4_—65.58 C_2_H_6_—0.78 C_3_H_8_—0.22 CO_2_—30.25 N_2_—3.18
	F3	60:40	20.771	CH_4_—56.21 C_2_H_6_—0.67 C_3_H_8_—0.19 CO_2_—40.21 N_2_—2.72

**Table 4:** Combustion conditions.

Fuel Type	Power Output, kW	Air Ratio (*λ*)	
		Without Li-Catalyst	With Li-Catalyst
F1	1.5	1.67	1.67
F2		1.77	1.77
F3		1.79	1.79

**Table 5:** Comparison of maximum temperatures within the porous medium, average CO and NOx concentrations.

Fuel Label	Without Li-Catalyst			With Li-Catalyst			Comparation	
	*t*_MAX_, °C	CO, ppm (vol)	NOx, ppm (vol)	*t*_MAX_, °C	CO, ppm (vol)	NOx, ppm (vol)	CO (%)	NOx (%)
F1	1229.30	71	12	1252.50	1	6	98.6	50.0
F2	1231.90	18	11	1260.10	5	5	72.2	54.5
F3	1202.10	15	4	1253.50	5	4	66.7	0.0

## Data Availability

Data are contained within the article.
